# Predicting mortality after transcatheter aortic valve replacement using preprocedural CT

**DOI:** 10.1038/s41598-024-63022-x

**Published:** 2024-05-31

**Authors:** David Brüggemann, Denis Cener, Nazar Kuzo, Shehab Anwer, Julia Kebernik, Matthias Eberhard, Hatem Alkadhi, Felix C. Tanner, Ender Konukoglu

**Affiliations:** 1 Computer Vision Laboratory, ETH Zurich, 8092 Zurich, Switzerland; 2 Department of Cardiology, University Heart Center, University Hospital Zurich, 8091 Zurich, Switzerland; 3 Institute for Diagnostic and Interventional Radiology, University Hospital Zurich, 8091 Zurich, Switzerland

**Keywords:** Interventional cardiology, Mathematics and computing

## Abstract

Transcatheter aortic valve replacement (TAVR) is a widely used intervention for patients with severe aortic stenosis. Identifying high-risk patients is crucial due to potential postprocedural complications. Currently, this involves manual clinical assessment and time-consuming radiological assessment of preprocedural computed tomography (CT) images by an expert radiologist. In this study, we introduce a probabilistic model that predicts post-TAVR mortality automatically using unprocessed, preprocedural CT and 25 baseline patient characteristics. The model utilizes CT volumes by automatically localizing and extracting a region of interest around the aortic root and ascending aorta. It then extracts task-specific features with a 3D deep neural network and integrates them with patient characteristics to perform outcome prediction. As missing measurements or even missing CT images are common in TAVR planning, the proposed model is designed with a probabilistic structure to allow for marginalization over such missing information. Our model demonstrates an AUROC of 0.725 for predicting all-cause mortality during postprocedure follow-up on a cohort of 1449 TAVR patients. This performance is on par with what can be achieved with lengthy radiological assessments performed by experts. Thus, these findings underscore the potential of the proposed model in automatically analyzing CT volumes and integrating them with patient characteristics for predicting mortality after TAVR.

## Introduction

Calcific aortic stenosis is a cardiovascular disease that is characterized by the thickening and calcification of the aortic valve, which can lead to inhibited leaflet motion and restriction of blood flow. It is the third-most frequent cardiovascular disease in developed countries, after coronary artery disease and systemic arterial hypertension^1^. If left untreated, it can lead to chest pain, loss of consciousness, and even death due to heart failure. Patients with severe aortic stenosis have three treatment options: surgical aortic valve replacement, transcatheter aortic valve replacement (TAVR), and medical therapy^2^. TAVR represents an alternative to open-heart surgery for patients deemed to be at high or prohibitive surgical risk due to its minimally invasive nature and shorter postprocedural hospital stays. However, for certain patients, TAVR has been associated with post-interventional complications, such as residual aortic regurgitation and mortality^3^. Identifying the patients who are likely to suffer from life-threatening complications remains a crucial clinical challenge.

Before an intervention, patients undergo a comprehensive assessment based on various factors, including their health status and medical history. An important component of this assessment is a computed tomography (CT) scan of the chest and abdomen. The CT scan is mainly used for valve sizing and access route evaluation. However, it also provides valuable information for TAVR risk stratification, such as the severity and distribution of calcification around the aortic valve^4^, coronary arteries^5^, and ascending aorta. The CT image is analyzed qualitatively or quantitatively, with manually extracted measurements that are believed to be linked to the procedural outcome. However, the need for a radiologist to extract these measurements makes the preprocedural assessment more time-consuming and expensive. Furthermore, it remains an open question how these measurements are best integrated into a TAVR risk prediction model. Deep neural networks (DNNs) could potentially help to address these challenges by extracting *optimal* image features automatically and combining them in *optimal* ways with other patient characteristics based on a set of training examples.

DNNs have demonstrated enormous potential for improving clinical workflows by automating repetitive and time-consuming tasks, such as the segmentation of anatomical structures^6^ and lesions^7^, and the detection of nodules^8^ and calcifications^9^. They have also been applied to more complex image-based prediction tasks, such as assessing disease risk^10^, phenotype^11^, and infarct tissue^12^. TAVR risk prediction is a particularly challenging task because the outcome is related not only to preprocedural CT and patient characteristics but also to the procedure itself. Nevertheless, it shares commonalities with other image-based prediction tasks, as there are visible changes in the images that are believed to be relevant to the outcome.

In this study, we introduce a probabilistic model to incorporate unprocessed, preprocedural CT images along with tabular baseline patient characteristics for TAVR risk prediction. Specifically, we focus on predicting all-cause mortality during the postprocedural follow-up period as a means of quantitative risk assessment. Informative features are extracted fully automatically from the CT images via a 3D DNN, requiring no manual image measurements by experts. Due to the structure of our model, we can avoid explicit imputation and instead use *marginalization* to deal with missing data, a common challenge in clinical settings. Both for training and inference, the required joint probabilities are calculated by marginalizing out the missing variables, i.e., summing or integrating over all possible values of the missing variables, to determine the marginal contribution of the non-missing data. Since we embed the CT images in a low-dimensional feature space via the DNN, missing CT images can be treated in the same manner: If the CT image is missing for a patient, it can be marginalized out analytically to calculate the joint probability of the non-missing data. Our main hypothesis is that our DNN-based method can extract *task-optimized* features from the images and yield accurate predictions while requiring no manual labor for integrating images in the preprocedural assessment process. To the best of our knowledge, this is the first study investigating the usefulness of unprocessed CT images for TAVR outcome prediction.

We evaluate our approach using a database that consists of preprocedural imaging and multiple non-imaging variables, as well as mortality information during the postprocedural follow-up, acquired from a set of patients who underwent TAVR. In addition to the preprocedural CT images, the database also contains multiple image-based measurements extracted by radiologists. In our experiments, we compare the prediction performance of manually-extracted measurements with DNN-extracted features, in combination with available non-imaging variables.

## TAVR data set

Data collection was performed in the context of a nationwide prospective TAVR registry (SwissTAVI Registry; ClinicalTrials.gov identifier, NCT01368250) and was approved by local and institutional review boards (KEK-Nr. 2013-0059). Written informed consent was obtained from all participants. For this single-center study, we performed a retrospective analysis of 1449 prospectively included participants with severe aortic stenosis undergoing nonenhanced and contrast-enhanced CT as part of their routine work-up for TAVR. The considered patient attributes can be split into four categories (see Table 1), of which the first three are baseline characteristics.

**Table 1:** Available patient characteristics and descriptive statistics within the study population. Parameters are the mean and standard deviation for continuous variables and the fraction of True instances for binary variables. LVOT: left ventricular outflow tract.

Variable	Type	Survivors, 813 (56.1%)		Non-survivors, 636 (43.9%)	
		Parameters	Missing	Parameters	Missing
Tabular baseline patient characteristics					
Age [y]	Cont.	79.8 ± 7.6	0.2%	82.2 ± 6.8	0.2%
Aortic regurgitation [severity score between 0 and 3]	Cont.	0.66 ± 0.70	13.0%	0.67 ± 0.70	21.1%
Aortic valve area [${\text {mm}^2}$]	Cont.	0.77 ± 0.20	12.2%	0.76 ± 0.21	20.6%
Body mass index [${\text {kg/m}^{2}}$]	Cont.	27.2 ± 4.8	0.4%	26.4 ± 4.8	0.2%
Cerebrovascular disease	Binary	0.18	0.6%	0.19	0.3%
Chronic obstructive pulmonary disease	Binary	0.13	1.0%	0.18	0.8%
Coronary artery bypass grafting	Binary	0.19	2.6%	0.21	0.6%
Coronary atheromatosis or stenosis	Binary	0.69	44.3%	0.78	15.6%
Creatinine [$\mu$ mol/L]	Cont.	99.7 ± 54.0	10.1%	121.6 ± 84.3	5.3%
Diabetes mellitus	Binary	0.23	2.1%	0.27	0.8%
Dyslipidemia	Binary	0.61	1.8%	0.51	1.7%
Family history of any cardiovascular disease	Binary	0.26	8.7%	0.33	2.7%
Glomerular filtration rate [mL/min/$\text {1.73m}^2$]	Cont.	59.7 ± 19.6	1.0%	53.5 ± 21.9	1.9%
Hemoglobin [g/L]	Cont.	124.2 ± 18.9	10.7%	118.0 ± 19.0	5.5%
Hypertension	Binary	0.80	1.5%	0.81	0.5%
Left ventricular ejection fraction [%]	Cont.	55.0 ± 12.8	9.2%	53.1 ± 13.9	18.2%
Male	Binary	0.52	0.5%	0.53	0.3%
Mean transaortic pressure gradient [mmHg]	Cont.	42.3 ± 15.7	9.0%	40.7 ± 16.1	17.6%
Mitral regurgitation [severity score between 0 and 3]	Cont.	0.92 ± 0.78	15.7%	1.14 ± 0.84	24.4%
Pacemaker at baseline	Binary	0.95	1.8%	0.89	8.3%
Peripheral artery disease	Binary	0.18	0.9%	0.27	0.3%
Previous cardiovascular interventions	Binary	0.23	3.6%	0.17	0.8%
Renal replacement or dialysis	Binary	0.01	11.4%	0.05	5.2%
Smoking status	Binary	0.29	39.6%	0.33	9.6%
Valve in valve	Binary	0.05	0%	0.02	0.2%
Preprocedural 3D torso CT image					
CT image	Cont.	–	58.1%	–	37.1%
CT image measurements					
Agatston score aortic valve	Cont.	2787.0 ± 1821.4	28.9%	2715.0 ± 1675.9	14.9%
Area-derived diameter of annulus incl. calcification [mm]	Cont.	24.2 ± 4.0	6.6%	24.6 ± 2.8	8.0%
Area of annulus incl. calcification [${\text {mm}^2}$]	Cont.	460.5 ± 97.5	6.6%	479.7 ± 107.0	8.0%
Calcification of ascending aorta	Cont.	1.9 ± 1.7	41.0%	1.9 ± 0.8	16.0%
Calcification of sinotubular junction	Cont.	1.1 ± 0.8	61.4%	1.1 ± 0.8	37.4%
Diameter of ascending aorta [mm]	Cont.	35.1 ± 4.3	6.0%	34.8 ± 4.2	7.7%
LVOT area [${\text {mm}^2}$]	Cont.	433.1 ± 111.6	6.9%	450.2 ± 117.9	7.2%
LVOT maximal diameter [mm]	Cont.	28.2 ± 3.4	5.7%	28.5 ± 3.9	7.2%
Maximal annulus diameter [mm]	Cont.	27.4 ± 3.0	6.4%	27.9 ± 3.1	8.0%
Maximal diameter of sinotubular junction [mm]	Cont.	29.2 ± 3.6	6.8%	29.3 ± 3.7	8.0%
Perimeter of annulus incl. calcification [mm]	Cont.	77.2 ± 8.4	6.5%	79.3 ± 8.7	8.0%
Sinus portion maximal diameter [mm]	Cont.	32.5 ± 4.0	7.0%	33.2 ± 3.9	7.9%
Volume of sinus valsalva [${\text {mm}^3}$]	Cont.	23.3 ± 9.7	45.1%	17.7 ± 7.2	36.5%
Volume score aortic valve	Cont.	1180.5 ± 926.8	57.7%	967.6 ± 736.8	37.3%
Volume score LVOT	Cont.	36.6 ± 85.7	13.2%	52.4 ± 108.7	10.1%
Outcome					
Death during follow-up	Binary	–	0%	–	0%

### Tabular baseline patient characteristics

The 25 tabular baseline patient characteristics listed in Table 1 were hand-picked from a larger set by medical experts. We purposely refrain from including any information about the interventional approach (e.g., access site, valve type), even though such information could arguably benefit the model prediction. Instead, we confine the tabular characteristics to clearly quantifiable baseline attributes.

### Preprocedural 3D torso CT images

For 741 patients (51%) a preprocedural 3D CT image of the torso is available. The field of view of CT scans varies substantially between patients: some images include the entire torso from the pelvic to the neck area, while others only contain the chest. Importantly, all CT scans cover the left ventricle, ascending aorta, and aortic arch.

The CT images show a large variability across different individuals, and not all the variability is necessarily relevant for predicting the outcome. Early experiments suggested that a 3D DNN struggles to extract meaningful patterns from such large-scale and heterogeneous input data. We believe this is due to large neural networks such as the one used in this study being prone to overfitting^13^. The network could still learn patterns given enough training data, however, we are limited by a small sample size in this study. Therefore, we chose to extract a task-relevant volumetric region of interest (ROI) and focus on this area to reduce the irrelevant inter-subject variability. It is naturally not feasible to know in advance all the ROIs relevant for a given outcome, thus we resort to clinical expertise to define an ROI around the aortic root and ascending aorta and extract it fully automatically as described below.

**Figure 1:**
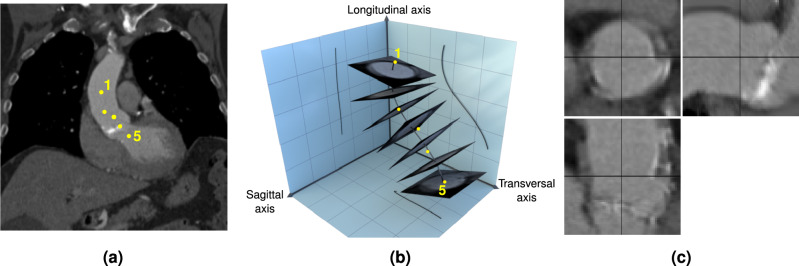
(**a**) Example image slice with oblique orientation showing the locations of the five aortic landmarks. The image slice is defined by the plane containing landmarks 1, 3, and 5. The dots for landmarks 2 and 4 are projections on that plane. (**b**) 3D schematic of the region of interest (ROI) extraction from the CT volume. The landmarks (yellow dots) are interpolated with a smoothing spline (black curve), from which orthogonal image slices are extracted. For visualization purposes, only seven slices are shown in this figure. The extracted image slices are simply stacked to obtain the ROI. (**c**) Cross-sectional views through the center of the ROI.

We use a deep learning-based approach to localize five landmarks on the center-line of the aorta in the CT images, starting from the aortic root and going through the ascending aorta as shown in Fig. 1a. These landmarks were chosen due to their proximity to the *device landing zone*, which covers the area around the aortic valve relevant for TAVR prosthesis stability and anchoring. For the landmark localization algorithm, we combine a reinforcement learning-based approach^14^ and a regression-based approach^15^. This two-stage procedure provides both the robustness and flexibility of the reinforcement learning-based method and the accuracy of the regression-based method. For brevity, we do not provide the details of the localization here as it is a re-implementation of existing methods^14,15^. The mean error of the localization is 4.1 mm compared to the manual placements of an experienced radiologist. We confirmed that exchanging the automatically and manually placed landmarks leads to no significant difference in downstream prediction performance.

Once the landmarks are localized, the ROI is extracted using four steps, visualized in Fig. 1b: (1) Interpolate the five landmarks with a cubic spline to obtain a center line through the aorta. (2) Define 64 intersection points evenly spaced along the spline. (3) At each intersection point, extract a 64 $\times$ 64 image slice from the image volume perpendicular to the spline. (4) Stack the image slices to obtain the final volumetric ROI. This final ROI of size 64 $\times$ 64 $\times$ 64 contains a “straightened” volume going from the aortic root through the ascending aorta. To account for inaccuracies in the landmark location prediction and increase the robustness of the extraction process, a cubic smoothing spline is used in step 1. Defining the orientation of the quadratic image slices as described in step 3 still leaves one degree of freedom remaining: a rotation of the slice around the axis of the spline at the intersection point. This degree of freedom is fixed by forcing one of the axes of the quadratic slice to be aligned with a projection of the sagittal axis on the slice. Fig. 1c shows three orthogonal center slices for an example ROI.

### CT image measurements

To facilitate a direct comparison between DNN-extracted image-based predictors and those extracted manually by medical experts, a radiologist specializing in cardiac imaging extracted 15 continuous valued measurements from the preprocedural CT images. The measurements were extracted either manually or using semi-automatic software and follow published guidelines and recommendations^16^. Not all measurements could be made for all the patients.

### Outcome

In this study, we define the outcome as all-cause mortality during the postprocedural follow-up period. Among survivors, the median follow-up duration is 1093 days, with the 5th and 95th percentiles at 366 days and 2683 days, respectively. Across all patients, the two output classes are fairly balanced: 44% of patients passed away during follow-up (class 1) and 56% survived (class 0).

## Probabilistic model

### Model definition

**Figure 2:**
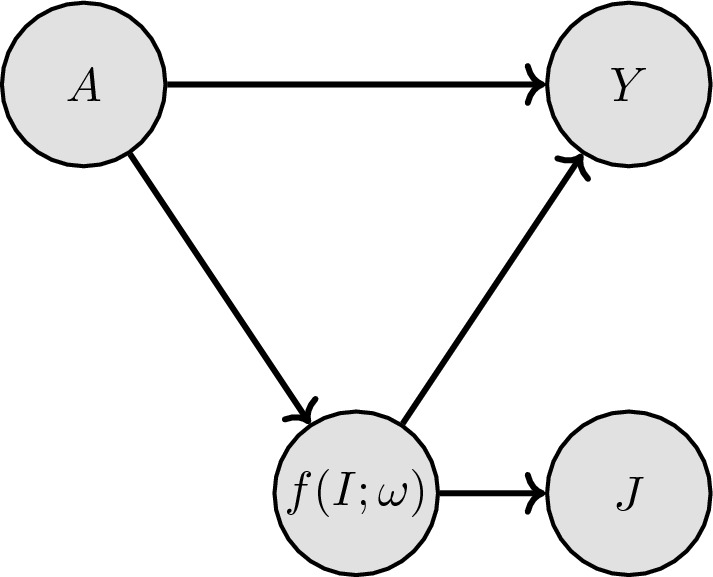
Directed acyclic graph expressing the conditional dependence structure of the TAVR data. *A* are the tabular characteristics, $f(I;\omega )$ are automatically extracted image features, *J* are manual image measurements, and *Y* is the outcome. Arrows indicate a dependency, e.g., *Y* only depends on *A* and $f(I;\omega )$.

We start by defining a graph that captures the assumed conditional dependence structure of our data. Based on clinical experience, we presume that the anatomy of the patient and pathological phenotypes captured in the CT image ROI, denoted as *I*, can influence the outcome, denoted as *Y*. We define a DNN-based feature extractor by $f(I;\omega )$– parameterized by $\omega$– which embeds such relevant anatomical features into a compact representation. Since the DNN-based feature representation has access to the entire image, we assume in the model that the representation $f(I;\omega )$ influences the manually extracted image measurements *J*, as changes in the underlying anatomy lead to changes in the image measurements. Finally, the tabular patient characteristics *A* (age, body mass index, smoking status, etc.) can influence both the representation $f(I;\omega )$ and the outcome *Y*. We thus arrive at the directed acyclic graph shown in Fig. 2, which outlines the structure of our probabilistic model. Importantly, our model leverages available manual image measurements *J* not as inputs, but as *auxiliary outputs*. *J* is not required for inference of *Y* if the DNN-based feature extractor’s outputs $f(I;\omega )$ are given, rather, is influenced by $f(I;\omega )$. As we will show, setting *J* as auxiliary output provides two opportunities: (i) when the feature extractor cannot be evaluated, e.g., if the unprocessed images are not available, available *J*’s can be used instead for inferring *Y*, and (ii) during training, *J* provides an additional signal to better train the DNN-based feature extractor as an *auxiliary task*.

The factorization of the joint probability of all involved variables can be written as

1 $$\begin{aligned} p(A, f(I; \omega ), J, Y) = p(Y | A, f(I; \omega )) \cdot p(J | f(I; \omega )) \cdot p(f(I; \omega ) | A) \cdot p(A). \end{aligned}$$

To facilitate closed-form expressions for the joint probability, which allows computational tractability, and to allow for data-efficient parameter estimation, we make the following assumptions: We assume that features within *A* and *J* are conditionally independent given $f(I;\omega )$. We model continuous features in *A* with Gaussian distributions and binary variables in *A* with Bernoulli distributions. The corresponding hyperparameters of *p*(*A*) are chosen (and fixed) to maximize the marginal likelihood of *A* for training observations. The link between the CT image and influencing patient factors is modeled with a Gaussian

2 $$\begin{aligned} p(f(I; \omega ) | A) = \mathscr {N}\left( f(I; \omega ); \beta ^T A, \sigma _I^2\textbf{I}_I \right) , \end{aligned}$$

with standard deviation $\sigma _I$ ($\textbf{I}_I$ denotes the identity matrix of appropriate dimensions) and model parameters $\beta$. A full covariance matrix could also be used for modeling, however, estimating the parameters of such a matrix would require a large number of training observations and a difficult optimization. Hence, we model the covariance with an isotropic covariance matrix. Similarly, the image measurements *J* are linearly linked to the image features with

3 $$\begin{aligned} p(J | f(I;\omega )) = \mathscr {N}(J; \phi ^T f(I;\omega ), \sigma _J^2\textbf{I}_J). \end{aligned}$$

Finally, we choose a logistic regression model to predict our binary outcome, where $\sigma (\cdot )$ denotes the sigmoid function.

4 $$\begin{aligned}&p(Y | A, f(I; \omega )) = \mu _Y ^ Y (1 - \mu _Y) ^ {1-Y} \end{aligned}$$

5 $$\begin{aligned}&\mu _Y = \sigma \left( \alpha _I^T \left( f(I; \omega ) - \beta ^T A\right) + \alpha _A^T A + b_Y \right) \end{aligned}$$

### Feature extraction with deep neural networks

To incorporate the CT volumes into the probabilistic model, we need to extract task-specific features from the ROIs via $f(I;\omega )$. Deep neural networks have proven effective in achieving such tasks, most prominently on 2D, but also on 3D data^17^. We thus use a 3D neural network as our feature extractor since the input CT images are volumetric. While any 3D neural network could be integrated into our probabilistic model, we specifically explore two pre-existing architectures in this work: (1) A 3D version of the seminal, convolutional ResNet-50^18^ architecture where all 2D convolutions are replaced by their 3D counterparts. (2) Swin UNETR^19^, a more recent, transformer-based architecture for 3D medical image analysis. We refer to the original publications^18,19^ for detailed descriptions of those networks.

3D ResNet-50 and Swin UNETR backbones embed a given input ROI into a feature vector with 2048 and 768 dimensions respectively. In the proposed approach, such large embedding sizes imply high parameter counts in the probabilistic model, i.e., $\beta$, $\phi$, $\alpha _I$, creating a risk of overfitting. To avoid this, we first reduce the feature dimensions to 16. The simplest way to achieve this would be to insert a fully connected layer, however, this would also introduce 2048 $\times$ 16 (resp. 768 $\times$ 16) additional parameters. Instead, we divide the backbone embedding dimensions, i.e., 2048 for the 3D ResNet-50 and 768 for the Swin UNETR, into 16 chunks, and compress each chunk to a single value via a linear transform. The number of additional parameters introduced by this transformation layer remains the same as the embedding dimension, i.e., 2048 and 768 for the ResNet and Swin UNETR, respectively. Consequently, we end up with an image representation $f(I;\omega )$ of size 16, which is further processed in the probabilistic model. $\omega$ encompasses all the learnable parameters of the 3D neural network and our additional compression layer.

It is a well-established fact that deep neural networks require large amounts of training data^20^. However, publicly available large-scale 3D medical data sets are rare in general, since medical data is much more expensive to collect, and 3D annotations are far more laborious. A large-scale data set for TAVR is not available to the best of our knowledge. Transfer learning from models pre-trained on natural images has been employed as a potential remedy^21^, however, the large domain gap between natural and medical images limits the effectiveness of this approach. More recently, researchers have compiled 3D medical data from several different sources to pre-train large-capacity models in a supervised^22^ or self-supervised^19^ way. The resulting pre-trained networks are shown to be highly data-efficient when fine-tuned to downstream medical image analysis tasks. We thus initialize our 3D ResNet-50 and Swin UNETR using those pre-trained weights^19,22^.

### Marginalization for missing data

For many patients in this study, one or more variables are missing. Missing data is a common scenario in clinical settings, and it can exacerbate the difficulty of predicting outcomes. Unrecorded variables can occur for several reasons, such as patient refusal to provide certain information, investigator or mechanical failure, or lack of resources or time to conduct certain investigations. Hereafter, we assume that the missing data is either *missing completely at random* or *missing at random*. Standard techniques to deal with an incomplete data set fulfilling this assumption include deletion and imputation. In deletion, observations with missing entries are discarded. In our case, this would eliminate 69.6% of all observations in our data set. With imputation, missing entries are estimated based on available data and are assumed to be given in the first place during model training. Since our model is fully probabilistic, we have the more principled option of marginalizing out variables that are not available for a given observation. For continuous variables, i.e., *I*, and possibly some *A* dimensions, this procedure for an observation *n* is

6 $$\begin{aligned} p(A_n, J_n, Y_n)&= \int p(A_n, J_n, f(I; \omega ), Y_n) dI \end{aligned}$$

7 $$\begin{aligned} p(A_n^{(\setminus k)}, J_n, f(I_n; \omega ), Y_n)&= \int p(A_n^{(\setminus k)}, A^{(k)}, J_n, f(I_n; \omega ), Y_n) dA^{(k)} \end{aligned}$$

where $\setminus k$ represents the set of all dimensions except *k*. Here, we assume that the image *I* is either complete or fully missing. Missing variables in *J* can be simply ignored, as they are output variables.

For binary variables, i.e., possibly some dimensions of *A*, the procedure is very similar, except with summations

8 $$\begin{aligned} p(A_n^{(\setminus k)}, J_n, f(I; \omega ), Y_n) = \sum _{\mathscr {A}^{(k)}} p(A_n^{(\setminus k)}, A^{(k)}, J_n, f(I_n; \omega ), Y_n), \end{aligned}$$

where $\mathscr {A}^{(k)}$ are the sets of possible values for the corresponding dimensions.

Through reasonable assumptions, we can derive analytical expressions for the joint probability in Eq. (1) for every possible combination of missing data in *A*, *J*, and *I*. Encountering the convolution of a Gaussian with a logistic sigmoid, we approximate the logistic sigmoid function with a scaled inverse probit function, an accurate and widely used approximation^23^. For more details, please refer to the supplementary material.

### Manual image measurements as auxiliary outputs

In this section, we highlight the interaction, emerging from the structure of our probabilistic model, between the manual image measurements *J* and the unprocessed image *I*. As shown in Eq. (3) and Fig. 2, we connect the DNN-based feature extractor’s outputs $f(I; \omega )$ to the manually determined image measurements *J* in our model. In this way, the image measurements *J* are leveraged as additional, auxiliary outputs, introducing *multi-task learning*^24^. The extra outputs can be considered as hints^25^, which induce a bias towards learning useful feature representation in the network $f(I; \omega )$. If applied to related tasks, this has been shown to improve sample efficiency and generalization for medical problems^26^ and beyond.

Importantly, by using them as auxiliary outputs instead of inputs, we remove the need for the measurements *J* for inference if we are given the unprocessed image *I*. In other words, if unprocessed images are given, the model does not need the manually extracted measurements for making a prediction. For a complete observation *n*, the joint probability of our model can be written as (see Eq. (1–5)):

9 $$\begin{aligned} p(A_n, f(I_n; \omega ), J_n, Y_n)&= \mu _{n}^{Y_n}(1 - \mu _{n})^{1 - Y_n} \cdot \mathscr {N}(J_n; \phi ^T f(I_n;\omega ), \sigma _J^2\textbf{I}_J) \cdot \mathscr {N}\left( f(I_n; \omega ); \beta ^T A_n, \sigma _I^2\textbf{I}_I \right) \cdot p(A_n) \end{aligned}$$

10 $$\begin{aligned} \mu _n&= \sigma \left( \alpha _I^T \left( f(I_n; \omega ) - \beta ^T A_n\right) + \alpha _A^T A_n + b_Y \right) \end{aligned}$$

Since the image measurements $J_n$ and outcome $Y_n$ are conditionally independent given the image $I_n$, the outcome prediction $\mu _n$ does not depend on $J_n$. So for inference with Eq. (10), we only need access to the tabular patient factors $A_n$ and the unprocessed image $I_n$. Now consider a scenario in which we are given an observation with some image measurements $J_n$ but a *missing* unprocessed image $I_n$. This can happen if the image cannot be shared between hospitals or is difficult to retrieve from the archiving system. Interestingly, the dependency structure of our model shifts in this case:

11 $$\begin{aligned} p(A_n, J_n, Y_n)&= \mu _{n,\setminus I}^{Y_n}(1 - \mu _{n,\setminus I})^{1 - Y_n} \cdot \mathscr {N}\left( J_n; \phi ^T \beta ^T A, \sigma _I^2\phi ^T\phi + \sigma _J^2\textbf{I}_J \right) \cdot p(A_n)\end{aligned}$$

12 $$\begin{aligned} \mu _{n,\setminus I}&= \sigma \left( \frac{\alpha _A^T A_n + b_Y + \sigma _I^2\alpha _I^T\phi \left( \sigma _I^2\phi ^T\phi + \sigma _J^2\textbf{I}_J\right) ^{-1}(J_n - \phi ^T \beta ^T A_n)}{\sqrt{1 + \frac{\pi }{8}\left( \sigma _I^2\alpha _I^T\alpha _I - \sigma _I^4\alpha _I^T\phi \left( \sigma _I^2\phi ^T\phi + \sigma _J^2\textbf{I}_J\right) ^{-1}\phi ^T\alpha _I\right) }}\right) \end{aligned}$$

Note that $\mu _{n,\setminus I}$– the prediction of the binary outcome $Y_n$– now directly depends on $J_n$ (compare Eq. (12) to Eq. (10)). By marginalizing out $I_n$, which influences both $J_n$ and $Y_n$, a causal link between the dependent variables is established. This hierarchy is provided through the probabilistic model. Crucially, Eq. (12) suggests that during inference, $J_n$ can be used to predict the outcome if (and only if) $I_n$ is missing. This underscores the flexibility of our model.

### Parameter learning

**Figure 3:**
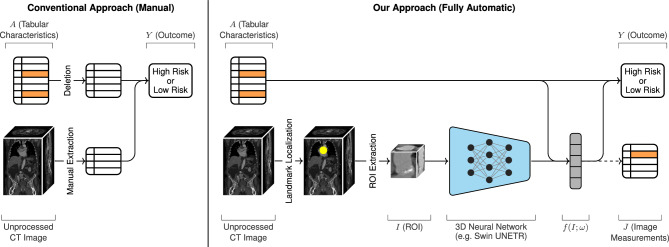
Overview of a conventional approach (left) and our automatic approach (right) for predicting TAVR outcome. In the conventional approach, radiologists manually extract measurements from the CT image, which are combined with the tabular characteristics *A* for risk assessment. Missing variables in *A* (shown in orange) are simply deleted. In our approach, task-specific image features $f(I;\omega )$ are automatically extracted from the CT volume through landmark localization, ROI extraction, and a 3D neural network. According to the hierarchy of our probabilistic model, the tabular patient factors *A* can also influence those image features $f(I;\omega )$. Missing values in *A* are marginalized. Both *A* and $f(I;\omega )$ are used to predict postprocedural mortality. In addition, our model leverages available manual image measurements *J* as auxiliary outputs to help guide the training of the network parameters $\omega$. The dashed arrow indicates that *J* is not required during inference.

Following common practice, we fit our model by maximizing its log-likelihood on training observations. Without missing variables, the log-likelihood for a set of *N* observations is:

13 $$\begin{aligned}{} & {} \sum _{n=1}^N \ln p(A_n, f(I_n;\omega ), J_n, Y_n) \propto \sum _{n=1}^N \biggl [ -\frac{1}{2} \sum _{j=0}^{d_J} \frac{\left( J_{n,j} - \phi ^Tf(I_n;\omega )\right) ^2}{\sigma _J^2} - d_J \ln (\sigma _J) \nonumber \\{} & {} \quad +Y_n \ln \mu _Y(I_n, A_n) + (1 - Y_n)\ln \left( 1 - \mu _Y(I_n, A_n)\right) \biggr ] \end{aligned}$$

In the above expression, constant terms are omitted since they do not affect the gradient. Analogous expressions for each missing data case are presented in the supplementary material. Crucially, this setup allows us to train the neural network parameters $\omega$ jointly with the parameters $\alpha _A, b_Y, \alpha _I, \beta , \sigma _I, \phi , \sigma _J$ of the probabilistic model. Our full model is shown in Fig. 3

## Results

To make better use of the available data, we evaluate the proposed models using 10-fold cross-validation. We use stratified cross-validation, as it has been shown to produce lower bias and variance in estimation^27^. For each fold, we split the data set into 80% training, 10% validation, and 10% test observations.

As a performance metric, we use the area under the ROC curve (AUROC). AUROC is used extensively in medical studies to assess binary classifications in biomedical sciences, such as the clinical classification of diseased from healthy patients, and to estimate the risk of adverse outcomes based on patients’ risk profiles^28^. Importantly, the AUROC provides a single-number performance metric for comparing the inherent validity of several diagnostic models, while avoiding defining a clinical decision criterion. Throughout the cross-validation experiments, we compute separate AUROC values for each fold and report the mean and standard error of the mean (SEM), in order to provide a measure of uncertainty about the estimate of AUROC. Implementation details and hyperparameters for the experiments can be found in the code at https://github.com/brdav/tavr.

### Comparison to alternative approaches

In Table 2 we present a performance comparison of our probabilistic model to six alternative approaches. The first two approaches estimate the patient risk based on the EuroSCORE II and the Society of Thoracic Surgeons (STS) score, respectively, using univariate logistic regression. The EuroSCORE II and STS score are two of the most influential cardiac surgery risk scores and have been widely used to risk-stratify patients for TAVR. The third and fourth approaches use the tabular variables in Table 1 for prediction, excluding (row 3) or including (row 4) the image measurements *J*. Missing variables are imputed via the sample mean. The approaches in rows 5 and 6 instead use marginalization to deal with missing values. Note that the fourth and sixth approaches are sophisticated multi-step approaches that involve 1) explicit measuring of relevant image features by medical experts, 2) either mean imputation or marginalization of missing variables, and 3) logistic regression. Table 2 shows that our probabilistic model, which unifies and automates all those steps, outperforms the risk-score-based and image-free models and performs on par with the image-based manual approaches. Importantly, our model only resorts to manual image measurements *J* when the unprocessed image *I* is missing (for 49% of patients in our data set). In those cases, it seamlessly marginalizes out the missing high-dimensional image input, which would not be possible with conventional data imputation. We present results using both the 3D ResNet-50^22^ and Swin UNETR^19^ as image feature extractors, whereby both networks perform similarly. Models with a risk within one standard error of the best model are boldfaced.

**Table 2:** AUROC for predicting death during follow-up after TAVR.

Method	Predictors	Missing data	AUROC $\uparrow$	
			mean	SEM
Logistic regression	EuroSCORE II	N/A	0.651	0.012
Logistic regression	STS score	N/A	0.646	0.007
Logistic regression	*A*	Mean imputation	0.689	0.011
Logistic regression	*A* and *J*	Mean imputation	**0.717**	0.011
Logistic regression	*A*	Marginalization	0.682	0.011
Logistic regression	*A* and *J*	Marginalization	**0.723**	0.010
Ours (3D ResNet-50^22^)	*A* and (*I* or *J*)	Marginalization	**0.722**	0.011
Ours (Swin UNETR^19^)	*A* and (*I* or *J*)	Marginalization	**0.725**	0.010

We report the mean and standard error of the mean (SEM) for 10-fold cross-validation.

*A* tabular patient factors, *I* unprocessed CT image, *J* CT measurements.

### Importance of auxiliary outputs

To highlight the importance of using the image measurements *J* as auxiliary outputs, we train a version of our probabilistic model that ignores *J*, i.e., by completely removing the component in Eq. (3) from the model during training and inference. This model (i) lacks the guidance facilitated by the auxiliary outputs during training, and (ii) cannot leverage the manual measurements to better marginalize missing image inputs (as shown in Eq. (12)). As a consequence, we observe a noticeable performance drop compared to the full model: Using the 3D ResNet-50 feature extractor, the performance difference $\Delta$AUROC is 0.018, whereas it is 0.013 using the Swin UNETR.

### Model analysis

We analyze the contribution of different (sets of) predictors to the predictive power of our probabilistic model. All results in this section are obtained from our full probabilistic model using a 3D ResNet-50 feature extractor. By withholding different (sets of) predictors during the evaluation of our model, it marginalizes out those predictors. The resulting performance drop indicates how important the withheld predictors are for the model’s performance.

Table 3 compares the value of the tabular patient factors *A*, the manual image measurements *J*, and the unprocessed images *I*. For example, in row 1, all predictors except *A* are withheld during evaluation. Using purely the image *I* as input is unfortunately not possible on our data set because it is completely missing for 49% of patients. As evidenced by comparing rows 1 and 3, using image-based predictors increases mean AUROC substantially. For the fair interpretation of the results in Table 3 it is important to note that our model (row 6) only uses the manual measurements *J* as predictors *when the unprocessed image I is unavailable* (for 49% of patients in our data set). However, in the case of rows 2 and 3, the image measurements *J* are used for *all* patients because the unprocessed image *I* is completely withheld. Comparing rows 3 and 6 thus shows that the performance of our model remains stable whether the automatic feature extraction is used for the 51% patients with an unprocessed image (row 6), or the manual measurements are used instead for all patients (row 3). This enables a tremendous increase in workflow efficiency for TAVR planning. Finally, the decreased performance in row 4 vs. row 6 shows that the image measurements *J* are indeed important for the effective marginalization of the missing unprocessed images. The scores within one standard error of the best result are boldfaced.

**Figure 4:**
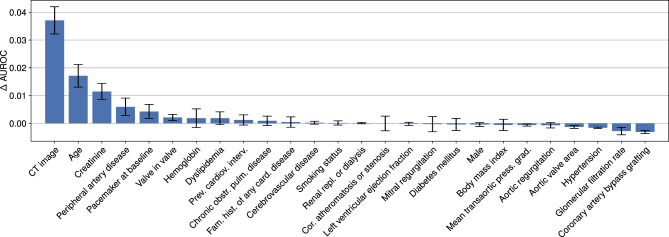
Predictors sorted by their importance for our probabilistic model. Withholding the “CT image” input (both the unprocessed image *I* and the measurements *J*) during evaluation leads to the largest drop in AUROC, indicating high importance. The “Age” predictor is second-most important after “CT image”. Bars indicate the standard error of the mean estimate.

We are also interested in assessing the importance of individual predictors for our probabilistic model. As shown in Fig. 4, our model relies predominantly on a fraction of the predictors. Withholding the image input (both the unprocessed image *I* and the measurements *J*) leads to a substantial mean AUROC drop of around 0.037.

**Table 3:** AUROC for predicting death during follow-up after TAVR with our probabilistic model, using different sets of predictors for inference.

	Predictors			AUROC $\uparrow$	
	*A* (tabular)	*J* (CT meas.)	*I* (CT image)	Mean	SEM
1	$\checkmark$			0.686	0.011
2	$\checkmark$			0.671	0.014
3	$\checkmark$	$\checkmark$		**0.725**	0.011
4	$\checkmark$		$\checkmark$	0.705	0.011
5		$\checkmark$	$\checkmark$	0.665	0.010
6	$\checkmark$	$\checkmark$	$\checkmark$	**0.722**	0.011

We report the mean and standard error of the mean (SEM) of 10-fold cross-validation.

## Discussion

In recent years, several works^29–33^ have explored the utility of machine learning models for TAVR outcome prediction. The approach presented in this paper differs in several aspects: (i) For simplicity, previous works^29–33^ exclusively use tabular features as predictors, foregoing the potential benefits of incorporating CT image information in the prediction. The model presented here addresses this shortcoming by leveraging unprocessed CT images through a powerful 3D DNN, circumventing the need for expensive, manual measurements of anatomical features, and instead extracting relevant features fully automatically from the CT. (ii) In previous studies, patients with missing variables were excluded^29,30,33^ or their missing variables were estimated based on the sample mean^32^. Our model’s generative probabilistic structure enables the marginalization of missing CT image inputs and patient variables, avoiding the need for explicit imputation. (iii) Previous works predict all-cause in-hospital mortality^30,32,33^, 1-year cardiovascular mortality^29^, and 5-year all-cause mortality^31^, whereas our approach focuses on predicting all-cause mortality during the follow-up. As the predicted clinical outcomes differ, reported performance values cannot be compared directly.

We evaluated our model in a cohort of 1449 TAVR patients. The results in Table 2 confirm that our automatic feature extraction can replace manually extracted image measurements without forfeiting prediction accuracy, which is an important and surprising point. The manually extracted features are complex measurements that integrate clinical experience and medical knowledge. However, extracting such features for every patient can create a bottleneck during preprocedural patient assessment: Extracting the measurements manually from a single image takes an expert radiologist between 10 and 15 minutes– depending on calcification severity. Our model delivers a prediction within 5 to 20 seconds on a consumer CPU– depending on the number of missing variables– allowing patient assessment with minimal manual labor. That said, an important part of the manually extracted features is their interpretability: Each manually extracted feature has a distinct clinical meaning. Such a meaning is conversely not available for the network-extracted features of our model: While post-hoc methods might help recover some explainability, those methods are approximative and would not reflect correct model behavior, which undermines their trust in clinical settings^34^.

There are also other clinically relevant limitations of our model. First, we provide the cross-validated generalization performance estimate on a single TAVR registry. All patients in this data set were treated in the same facility over around eight years. Furthermore, the data set is naturally biased towards high-risk patients, since only those patients were prescribed TAVR. Before using the presented model to guide treatment decisions, its performance should thus be evaluated on a separate, independent patient registry. Second, the proposed probabilistic model is built on the assumption of predictor independence. As such, the model could operate sub-optimally for predictor sets with highly correlated variables. However, this can be easily avoided by filtering (or transforming) the predictor set accordingly, as was done in this work. Third, although AUROC represents a good general metric for comparing clinical prediction models, it has limitations. Estimating a model’s performance only based on AUROC can be misleading. For instance, a model might have a very high true positive rate at clinically unacceptable levels of false positive rate and perform poorly in clinically relevant ranges^35^. Such a model could still score a high AUROC. Before clinical application, an operation range should thus be set by defining a cut-off for classifying patients as either high- or low-risk. A cut-off point is difficult to define: it requires the balancing of potential benefits and harms, which can be subjective. However, with a fixed cut-off, the model could be evaluated more directly in terms of specificity and sensitivity. Finally, a more comprehensive measure of TAVR success should incorporate not only mortality but also the patient’s quality of life and functional level after the procedure. This could provide a clinically more useful estimate of TAVR risk^36^. Additionally, there may be other important predictors of TAVR risk that were not collected in the examined TAVR registry, e.g., atrial fibrillation. However, our model does not necessitate structural modifications to accommodate additional variables or predict different outcomes. If a TAVR registry with this information becomes available, our model could be fit analogously.

## Supplementary Information

Supplementary Information.

## Data Availability

The data that support the findings of this study are part of the SwissTAVI Registry (ClinicalTrials.gov identifier: NCT01368250). Data is available on reasonable request from the corresponding author. Please note that sharing data is bound to institutional and national regulations and their process needs to be followed for sharing data.
