# Author Correction: Predicting mortality after transcatheter aortic valve replacement using preprocedural CT

**DOI:** 10.1038/s41598-025-94409-z

**Published:** 2025-03-26

**Authors:** David Brüggemann, Denis Cener, Nazar Kuzo, Shehab Anwer, Julia Kebernik, Matthias Eberhard, Hatem Alkadhi, Felix C. Tanner, Ender Konukoglu

**Affiliations:** 1 Computer Vision Laboratory, ETH Zurich, 8092 Zurich, Switzerland; 2 Department of Cardiology, University Heart Center, University Hospital Zurich, 8091 Zurich, Switzerland; 3 Institute for Diagnostic and Interventional Radiology, University Hospital Zurich, 8091 Zurich, Switzerland

Correction to: *Scientific Reports* 10.1038/s41598-024-63022-x, published online 31 May 2024

Denis Cener was omitted from the author list in the original version of this Article.

The Author Contributions section now reads:

“E.K., F.C.T., and H.A. contributed to the conception and design of the study. F.C.T., J.K., M.E., N.Z., and S.A. gathered, cleaned, and prepared the data for this study. D.B. and E.K. developed the data processing techniques and the probabilistic model. D.B. and D.C. implemented the model and conducted the experiments. D.B. and E.K. drafted the manuscript. All authors interpreted the data and revised the manuscript.”

The original Article has been corrected.

