# Supplementary material for: Predicting mortality after transcatheter aortic valve replacement using preprocedural CT
Source: Sci Rep. 2024 May 31;14:12526. doi: 10.1038/s41598-024-63022-x (PMC11143216; doi:10.1038/s41598-024-63022-x)
Supplement: Supplementary file 1 — Supplementary Information. [file 41598_2024_63022_MOESM1_ESM.pdf]

Predicting Mortality after Transcatheter Aortic Valve Replacement using Preprocedural CT: Supplementary Material

Marginalization for Binary Data

This case pertains to missing entries in the binary variables of A. In this case, marginalization involves summation over possible alternatives. As we only encounter binary variables, this simply entails summing over the cases when the missing variables are either 0 or 1. Note that this amounts to 2^k cases to sum over in case k binary variables are missing for a given instance. For an observation n the joint probability can be written as

$$p(A_n^{(\backslash k)}, f(I_n;\omega), J_n, Y_n) = p(A_n^{(\backslash k)}) p(J_n|f(I_n;w)) \sum_{\mathscr{A}^{(k)}} \left(p(A^{(k)}) p(f(I_n;w)|A_n^{(\backslash k)}, A^{(k)}) p(Y_n|f(I_n;w), A_n^{(\backslash k)}, A^{(k)}) \right), \quad (1)$$

where the superscript $(\backslash k)$ denotes non-missing dimensions and (k) denotes the missing dimensions. $\mathscr{A}^{(k)}$ are the sets of possible values for the missing dimensions. Since we have binary variables we sum over all possible binary codes of length k.

As we train our network via log-likelihood maximization, the objective to maximize is:

$$\ln p(A_n^{(\backslash k)}, f(I_n;\omega), J_n, Y_n) = \ln p(A_n^{(\backslash k)}) + \ln p(J_n|f(I_n;w)) + \ln \sum_{\mathscr{A}^{(k)}} \left(p(A^{(k)}) p(f(I_n;w)|A_n^{(\backslash k)}, A^{(k)}) p(Y_n|f(I_n;w), A_n^{(\backslash k)}, A^{(k)}) \right) \quad (2)$$

To avoid under- or overflow during loss computation, we use a numerically stable implementation of log-sum-exp to compute the last term. If some continuous variables are missing as well, the expressions derived in the next section can be combined with Eq. (2).

Marginalization for Continuous Data

There are three possible subcases: Missing data in A, missing image I, and both of the above. For brevity, we only provide the full derivation for the case of missing data in A, and directly write the log-likelihood for the other two cases. Since the derivations are similar, the following derivation can be used as a prototype for the other two cases.

Missing Data in A

This is the case when image I is given but some entries in A are missing. We can write the joint probability as

$$p(A^{(\backslash k)}, f(I;\omega), J, Y) = p(A^{(\backslash k)}) p(J|f(I;w)) \int p(A^{(k)}) p(f(I;w)|A^{(\backslash k)}, A^{(k)}) p(Y|f(I;w), A^{(\backslash k)}, A^{(k)}) dA^{(k)}, \quad (3)$$

where the superscript $(\backslash k)$ denotes non-missing dimensions and (k) denotes missing dimensions in A. As we will see, we only need to derive the result for the case $Y = 1$. Observe that

$$\begin{aligned} p(Y=1|f(I;w), A) &= \sigma\left(\alpha_I^T \left(f(I;\omega) - \beta^T A\right) + \alpha_A^T A + b_Y\right) \\ &= \sigma\left((\alpha_A - \beta\alpha_I)^T A + \alpha_I^T f(I;\omega) + b_Y\right). \end{aligned} \quad (4)$$

We split $(\alpha_A - \beta\alpha_I) = \begin{bmatrix} \gamma^{(\backslash k)} \\ \gamma^{(k)} \end{bmatrix}$, where $\gamma^{(\backslash k)}$ multiplies non-missing dimensions in A and $\gamma^{(k)}$ multiplies missing dimensions. As a result we obtain

$$\begin{aligned} p(Y=1|f(I;w), A^{(\backslash k)}, A^{(k)}) &= \sigma\left(\gamma^{(k)T} A^{(k)} + \gamma^{(\backslash k)T} A^{(\backslash k)} + \alpha_I^T f(I;\omega) + b_Y\right) \\ &= \int \sigma(\xi)\, \delta\left(\xi - \left(\gamma^{(k)T} A^{(k)} + \gamma^{(\backslash k)T} A^{(\backslash k)} + \alpha_I^T f(I;\omega) + b_Y\right)\right) d\xi, \end{aligned} \quad (5)$$

where we introduce a new variable ξ in the second line by making use of Dirac's delta $\delta(\cdot)$. We do the same for

$$\begin{aligned} p(f(I;w)|A^{(\backslash k)}, A^{(k)}) &= \mathscr{N}\left(f(I;\omega); \beta^{(k)T} A^{(k)} + \beta^{(\backslash k)T} A^{(\backslash k)}, \sigma_I^2 \mathbf{I}_I\right) \\ &= \int \mathscr{N}\left(f(I;\omega); \eta, \sigma_I^2 \mathbf{I}_I\right) \delta\left(\eta - \left(\beta^{(k)T} A^{(k)} + \beta^{(\backslash k)T} A^{(\backslash k)}\right)\right) d\eta, \end{aligned} \quad (6)$$

where we split $\beta = \begin{bmatrix} \beta^{(\backslash k)} \\ \beta^{(k)} \end{bmatrix}$ and we introduced the new variable η. Going back to Eq. (3), we can now write out the integral on the right-hand side by plugging in Eq. (5) and Eq. (6) and changing the order of integration.

$$\int \sigma(\xi) \int \mathcal{N}\left(f(I;\omega);\eta,\sigma_I^2\mathbf{I}_I\right) \int \mathcal{N}\left(A^{(k)};\mu_{A^{(k)}},\sigma^2_{A^{(k)}}\mathbf{I}_{A^{(k)}}\right)\delta\left(\eta-\left(\beta^{(k)T}A^{(k)}+\beta^{(\backslash k)T}A^{(\backslash k)}\right)\right)$$
$$\delta\left(\xi-\left(\gamma^{(k)T}A^{(k)}+\gamma^{(\backslash k)T}A^{(\backslash k)}+\alpha_I^T f(I;\omega)+b_Y\right)\right)dA^{(k)}d\eta d\xi \quad (7)$$

Observe that the innermost integral yields another Gaussian distribution: The Dirac's delta terms constrain $A^{(k)}$ to a linear subspace. The integral consequently integrates over all directions within the subspace, yielding a marginal distribution over the directions orthogonal to it. We know that the marginal of a Gaussian is another Gaussian, whose mean and variance we can determine by computing its first and second moments.

$$p\left(\xi,\eta|f(I;\omega),A^{(\backslash k)}\right)=\mathcal{N}\left(\begin{bmatrix}\xi\\ \eta\end{bmatrix};\begin{bmatrix}\mu_{\xi|f(I;\omega),A^{(\backslash k)}}\\ \mu_{\eta|f(I;\omega),A^{(\backslash k)}}\end{bmatrix},\begin{bmatrix}\Sigma_{\xi|f(I;\omega),A^{(\backslash k)}} & \Sigma_{\xi,\eta|f(I;\omega),A^{(\backslash k)}}\\ \Sigma_{\eta,\xi|f(I;\omega),A^{(\backslash k)}} & \Sigma_{\eta|f(I;\omega),A^{(\backslash k)}}\end{bmatrix}\right) \quad (8)$$
$$\mu_{\xi|f(I;\omega),A^{(\backslash k)}}=\gamma^{(k)T}\mu_{A^{(k)}}+\gamma^{(\backslash k)T}A^{(\backslash k)}+\alpha_I^T f(I;\omega)+b_Y \quad (9)$$
$$\mu_{\eta|f(I;\omega),A^{(\backslash k)}}=\beta^{(k)T}\mu_{A^{(k)}}+\beta^{(\backslash k)T}A^{(\backslash k)} \quad (10)$$
$$\Sigma_{\xi|f(I;\omega),A^{(\backslash k)}}=\gamma^{(k)T}\sigma^2_{A^{(k)}}\mathbf{I}_{A^{(k)}}\gamma^{(k)} \quad (11)$$
$$\Sigma_{\xi,\eta|f(I;\omega),A^{(\backslash k)}}=\gamma^{(k)T}\sigma^2_{A^{(k)}}\mathbf{I}_{A^{(k)}}\beta^{(k)} \quad (12)$$
$$\Sigma_{\eta,\xi|f(I;\omega),A^{(\backslash k)}}=\beta^{(k)T}\sigma^2_{A^{(k)}}\mathbf{I}_{A^{(k)}}\gamma^{(k)} \quad (13)$$
$$\Sigma_{\eta|f(I;\omega),A^{(\backslash k)}}=\beta^{(k)T}\sigma^2_{A^{(k)}}\mathbf{I}_{A^{(k)}}\beta^{(k)} \quad (14)$$

Note that we can back-substitute γ and β by defining $\mu_{A'} = \begin{bmatrix} A^{(\backslash k)} \\ \mu_{A^{(k)}} \end{bmatrix}$ and $\Sigma_{A'} = \begin{bmatrix} \mathbf{0}_{A^{(\backslash k)}} & \mathbf{0}_{A^{(\backslash k)},A^{(k)}} \\ \mathbf{0}_{A^{(k)},A^{(\backslash k)}} & \sigma^2_{A^{(k)}}\mathbf{I}_{A^{(k)}} \end{bmatrix}$. In other words, we can treat non-missing features in A analogous to the missing features, setting the mean equal to their actual value and the standard deviation to zero.

$$\mu_{\xi|f(I;\omega),A^{(\backslash k)}}=\gamma^T\mu_{A'}+\alpha_I^T f(I;\omega)+b_Y \quad (15)$$
$$\mu_{\eta|f(I;\omega),A^{(\backslash k)}}=\beta^T\mu_{A'} \quad (16)$$
$$\Sigma_{\xi|f(I;\omega),A^{(\backslash k)}}=\gamma^T\Sigma_{A'}\gamma \quad (17)$$
$$\Sigma_{\xi,\eta|f(I;\omega),A^{(\backslash k)}}=\gamma^T\Sigma_{A'}\beta \quad (18)$$
$$\Sigma_{\eta,\xi|f(I;\omega),A^{(\backslash k)}}=\beta^T\Sigma_{A'}\gamma \quad (19)$$
$$\Sigma_{\eta|f(I;\omega),A^{(\backslash k)}}=\beta^T\Sigma_{A'}\beta \quad (20)$$

Note that $p\left(\eta|f(I;\omega),A^{(\backslash k)}\right)=p\left(\eta|A^{(\backslash k)}\right)$. Moving on to the middle integral in Eq. (7), we observe that it is a convolution of Gaussians. To compute it, we first compute the joint distribution by refactoring the terms multiple times:

$$\int p(f(I;\omega)|\eta)\,p\left(\xi,\eta|f(I;\omega),A^{(\backslash k)}\right)d\eta=\int p(f(I;\omega)|\eta)\,p\left(\xi|\eta,f(I;\omega),A^{(\backslash k)}\right)p\left(\eta|A^{(\backslash k)}\right)d\eta \quad (21)$$
$$=\int p\left(\xi,f(I;\omega)|\eta,A^{(\backslash k)}\right)p\left(\eta|A^{(\backslash k)}\right)d\eta \quad (22)$$
$$=\int p\left(\xi,f(I;\omega),\eta|A^{(\backslash k)}\right)d\eta \quad (23)$$

In each refactoring step, we can use the general equations for the conditional distribution of a Gaussian distribution (e.g., see Eq. (2.81) and (2.82) in Bishop[1]) to read out the joint or conditionals. According to Eq. (21) we first factorize Eq. (8).

$$p(\xi,\eta|f(I;\omega),A^{(\backslash k)}) = p(\xi|\eta,f(I;\omega),A^{(\backslash k)})p(\eta|A^{(\backslash k)})$$
$$= \mathcal{N}\left(\xi;\mu_{\xi|\eta,f(I;\omega),A^{(\backslash k)}},\Sigma_{\xi|\eta,f(I;\omega),A^{(\backslash k)}}\right)\mathcal{N}\left(\eta;\beta^T\mu_{A'},\beta^T\Sigma_{A'}\beta\right) \tag{24}$$
$$\mu_{\xi|\eta,f(I;\omega),A^{(\backslash k)}} = \gamma^T\mu_{A'} + \alpha_I^T f(I;\omega) + b_Y + \gamma^T\Sigma_{A'}\beta\left(\beta^T\Sigma_{A'}\beta\right)^{-1}\left(\eta - \beta^T\mu_{A'}\right) \tag{25}$$
$$\Sigma_{\xi|\eta,f(I_n;\omega),A^{(\backslash k)}} = \gamma^T\Sigma_{A'}\gamma - \gamma^T\Sigma_{A'}\beta\left(\beta^T\Sigma_{A'}\beta\right)^{-1}\beta^T\Sigma_{A'}\gamma \tag{26}$$

Next, we compute $p\left(\xi,f(I;\omega)|\eta,A^{(\backslash k)}\right)$ in Eq. (22).

$$p\left(\xi,f(I;\omega)|\eta,A^{(\backslash k)}\right) = \mathcal{N}\left(\begin{bmatrix}\xi\\ f(I;\omega)\end{bmatrix};\begin{bmatrix}\mu_{\xi|\eta,A^{(\backslash k)}}\\ \mu_{f(I;\omega)|\eta,A^{(\backslash k)}}\end{bmatrix},\begin{bmatrix}\Sigma_{\xi|\eta,A^{(\backslash k)}} & \Sigma_{\xi,f(I;\omega)|\eta,A^{(\backslash k)}}\\ \Sigma_{f(I;\omega),\xi|\eta,A^{(\backslash k)}} & \Sigma_{f(I;\omega)|\eta,A^{(\backslash k)}}\end{bmatrix}\right) \tag{27}$$
$$\mu_{\xi|\eta,A^{(\backslash k)}} = \left(\gamma^T - \gamma^T\Sigma_{A'}\beta\left(\beta^T\Sigma_{A'}\beta\right)^{-1}\beta^T\right)\mu_{A'} + b_Y + \left(\alpha_I^T + \gamma^T\Sigma_{A'}\beta\left(\beta^T\Sigma_{A'}\beta\right)^{-1}\right)\eta \tag{28}$$
$$\mu_{f(I;\omega)|\eta,A^{(\backslash k)}} = \eta \tag{29}$$
$$\Sigma_{\xi|\eta,A^{(\backslash k)}} = \gamma^T\Sigma_{A'}\gamma - \gamma^T\Sigma_{A'}\beta\left(\beta^T\Sigma_{A'}\beta\right)^{-1}\beta^T\Sigma_{A'}\gamma + \sigma_I^2\alpha_I^T\alpha_I \tag{30}$$
$$\Sigma_{\xi,f(I;\omega)|\eta,A^{(\backslash k)}} = \sigma_I^2\alpha_I^T \tag{31}$$
$$\Sigma_{f(I;\omega),\xi|\eta,A^{(\backslash k)}} = \sigma_I^2\alpha_I \tag{32}$$
$$\Sigma_{f(I;\omega)|\eta,A^{(\backslash k)}} = \sigma_I^2\mathbf{I}_I \tag{33}$$

Finally, we substitute $\xi' = \begin{bmatrix}\xi\\ f(I;\omega)\end{bmatrix}$ and compute the joint distribution $p\left(\xi',\eta|A^{(\backslash k)}\right)$ in Eq. (23).

$$p\left(\xi',\eta|A^{(\backslash k)}\right) = \mathcal{N}\left(\begin{bmatrix}\xi'\\ \eta\end{bmatrix};\begin{bmatrix}\mu_{\xi'|A^{(\backslash k)}}\\ \mu_{\eta|A^{(\backslash k)}}\end{bmatrix},\begin{bmatrix}\Sigma_{\xi'|A^{(\backslash k)}} & \Sigma_{\xi',\eta|A^{(\backslash k)}}\\ \Sigma_{\eta,\xi'|A^{(\backslash k)}} & \Sigma_{\eta|A^{(\backslash k)}}\end{bmatrix}\right) \tag{34}$$
$$\mu_{\xi'|A^{(\backslash k)}} = \begin{bmatrix}\left(\alpha_I^T\beta^T + \gamma^T\right)\mu_{A'} + b_Y\\ \beta^T\mu_{A'}\end{bmatrix} \tag{35}$$
$$\mu_{\eta|A^{(\backslash k)}} = \beta^T\mu_{A'} \tag{36}$$
$$\Sigma_{\xi'|A^{(\backslash k)}} = \begin{bmatrix}\left(\alpha_I^T\beta^T+\gamma^T\right)\Sigma_{A'}\beta\alpha_I + \left(\alpha_I^T\beta^T+\gamma^T\right)\Sigma_{A'}\gamma + \sigma_I^2\alpha_I^T\alpha_I & \left(\alpha_I^T\beta^T+\gamma^T\right)\Sigma_{A'}\beta + \sigma_I^2\alpha_I^T\\ \beta^T\Sigma_{A'}\left(\beta\alpha_I+\gamma\right) + \sigma_I^2\alpha_I & \beta^T\Sigma_{A'}\beta + \sigma_I^2\mathbf{I}_I\end{bmatrix} \tag{37}$$
$$\Sigma_{\xi',\eta|A^{(\backslash k)}} = \begin{bmatrix}\left(\alpha_I^T\beta^T+\gamma^T\right)\Sigma_{A'}\beta\\ \beta^T\Sigma_{A'}\beta\end{bmatrix} \tag{38}$$
$$\Sigma_{\eta,\xi'|A^{(\backslash k)}} = \begin{bmatrix}\beta^T\Sigma_{A'}\left(\beta\alpha_I+\gamma\right) & \beta^T\Sigma_{A'}\beta\end{bmatrix} \tag{39}$$
$$\Sigma_{\eta|A^{(\backslash k)}} = \beta^T\Sigma_{A'}\beta \tag{40}$$

We can thus determine the middle integral in Eq. (7) by reading out the marginal

$$p\left(\xi,f(I;\omega)|A^{(\backslash k)}\right) = \mathcal{N}\left(\begin{bmatrix}\xi\\ f(I;\omega)\end{bmatrix};\mu_{\xi'|A^{(\backslash k)}},\Sigma_{\xi'|A^{(\backslash k)}}\right). \tag{41}$$

The outer integral in Eq. (7) takes the form

$$\int\sigma(\xi)p\left(\xi,f(I;\omega)|A^{(\backslash k)}\right)d\xi = p\left(f(I;\omega)|A^{(\backslash k)}\right)\int\sigma(\xi)p\left(\xi|f(I;\omega),A^{(\backslash k)}\right)d\xi, \tag{42}$$

where we again factorize the joint distribution using the expressions for the conditional distribution of a Gaussian:

$$p\left(f(I;\omega)|A^{(\backslash k)}\right) = \mathcal{N}\left(f(I;\omega);\beta^T\mu_{A'},\beta^T\Sigma_{A'}\beta+\sigma_I^2\mathbf{I}_I\right) \tag{43}$$

$$p\left(\xi|f(I;\omega),A^{(\backslash k)}\right) = \mathcal{N}\left(\xi;\mu'_{\backslash A^{(k)}},\sigma'^2_{\backslash A^{(k)}}\right) \tag{44}$$

$$\mu'_{\backslash A^{(k)}} = \left(\alpha_I^T\beta^T+\gamma^T\right)\mu_{A'}+b_Y+\left(\left(\alpha_I^T\beta^T+\gamma^T\right)\Sigma_{A'}\beta+\sigma_I^2\alpha_I^T\right)\left(\beta^T\Sigma_{A'}\beta+\sigma_I^2\mathbf{I}_I\right)^{-1}\left(f(I;\omega)-\beta^T\mu_{A'}\right) \tag{45}$$

$$\begin{aligned}\sigma'^2_{\backslash A^{(k)}} = &\left(\alpha_I^T\beta^T+\gamma^T\right)\Sigma_{A'}\beta\alpha_I+\left(\alpha_I^T\beta^T+\gamma^T\right)\Sigma_{A'}\gamma+\sigma_I^2\alpha_I^T\alpha_I\\ &-\left(\left(\alpha_I^T\beta^T+\gamma^T\right)\Sigma_{A'}\beta+\sigma_I^2\alpha_I^T\right)\left(\beta^T\Sigma_{A'}\beta+\sigma_I^2\mathbf{I}_I\right)^{-1}\left(\beta^T\Sigma_{A'}\left(\beta\alpha_I+\gamma\right)+\sigma_I^2\alpha_I\right)\end{aligned} \tag{46}$$

The integral on the right-hand side of Eq. (42) is the convolution of a Gaussian with a logistic sigmoid. To obtain an analytical expression, we approximate the sigmoid function $\sigma(\xi)$ with a scaled inverse probit function $\Phi\left(\sqrt{\frac{\pi}{8}}\xi\right)$[1].

$$\int\sigma(\xi)\mathcal{N}\left(\xi;\mu'_{\backslash A^{(k)}},\sigma'^2_{\backslash A^{(k)}}\right)d\xi\approx\int\Phi\left(\sqrt{\frac{\pi}{8}}\xi\right)\mathcal{N}\left(\xi;\mu'_{\backslash A^{(k)}},\sigma'^2_{\backslash A^{(k)}}\right)d\xi=\Phi\left(\frac{\sqrt{\frac{\pi}{8}}\mu'_{\backslash A^{(k)}}}{\sqrt{1+\frac{\pi}{8}\sigma'^2_{\backslash A^{(k)}}}}\right)\approx\sigma\left(\frac{\mu'_{\backslash A^{(k)}}}{\sqrt{1+\frac{\pi}{8}\sigma'^2_{\backslash A^{(k)}}}}\right) \tag{47}$$

We can now write out the full log-likelihood for one observation n. Note that we back-substituted $(\alpha_A-\beta\alpha_I)=\gamma$ into all equations.

$$\begin{aligned}\ln p(A_n^{(\backslash k)},J_n,f(I_n;\omega),Y_n) &= \ln\int p(A_n^{(\backslash k)},A^{(k)},J_n,f(I_n;\omega),Y_n)dA^{(k)}\\ &= \ln p(J_n|f(I_n;\omega))+\ln p(A_n^{(\backslash k)})+\ln\mu_{n,\backslash A^{(k)}}^{Y_n}(1-\mu_{n,\backslash A^{(k)}})^{1-Y_n}\\ &\quad+\ln\mathcal{N}\left(f(I_n;\omega);\beta^T\mu_{A'},\beta^T\Sigma_{A'}\beta+\sigma_I^2\mathbf{I}_I\right)\end{aligned} \tag{48}$$

$$\mu_{n,\backslash A^{(k)}} = \sigma\left(\frac{\mu'_{n,\backslash A^{(k)}}}{\sqrt{1+\frac{\pi}{8}\sigma'^2_{n,\backslash A^{(k)}}}}\right) \tag{49}$$

$$\mu'_{n,\backslash A^{(k)}} = \alpha_A^T\mu_{A'}+b_Y+\left(\alpha_A^T\Sigma_{A'}\beta+\sigma_I^2\alpha_I^T\right)\left(\beta^T\Sigma_{A'}\beta+\sigma_I^2\mathbf{I}_I\right)^{-1}\left(f(I;\omega)-\beta^T\mu_{A'}\right) \tag{50}$$

$$\sigma'^2_{n,\backslash A^{(k)}} = \alpha_A^T\Sigma_{A'}\alpha_A+\sigma_I^2\alpha_I^T\alpha_I-\left(\alpha_A^T\Sigma_{A'}\beta+\sigma_I^2\alpha_I^T\right)\left(\beta^T\Sigma_{A'}\beta+\sigma_I^2\mathbf{I}_I\right)^{-1}\left(\beta^T\Sigma_{A'}\alpha_A+\sigma_I^2\alpha_I\right) \tag{51}$$

where, as defined earlier, $\mu_{A'}=\begin{bmatrix}A_n^{(\backslash k)}\\ \mu_{A^{(k)}}\end{bmatrix}$ and $\Sigma_{A'}=\begin{bmatrix}\mathbf{0}_{A^{(\backslash k)}} & \mathbf{0}_{A^{(\backslash k)},A^{(k)}}\\ \mathbf{0}_{A^{(k)},A^{(\backslash k)}} & \sigma^2_{A^{(k)}}\mathbf{I}_{A^{(k)}}\end{bmatrix}$.

Missing Image I

This is the case when image I is missing and A is complete. We provide directly the log-likelihood for one observation n.

$$\begin{aligned}\ln p(A_n,J_n,Y_n) &= \ln\int p(A_n,J_n,f(I;\omega),Y_n)dI\\ &= \ln p(A_n)+\ln\mu_{n,\backslash I}^{Y_n}(1-\mu_{n,\backslash I})^{1-Y_n}+\ln\mathcal{N}\left(J_n;\phi^T\beta^TA,\sigma_I^2\phi^T\phi+\sigma_J^2\mathbf{I}_J\right)\end{aligned} \tag{52}$$

$$\mu_{n,\backslash I} = \sigma\left(\frac{\mu'_{n,\backslash I}}{\sqrt{1+\frac{\pi}{8}\sigma'^2_{n,\backslash I}}}\right) \tag{53}$$

$$\mu'_{n,\backslash I} = \alpha_A^TA+b_Y+\sigma_I^2\alpha_I^T\phi\left(\sigma_I^2\phi^T\phi+\sigma_J^2\mathbf{I}_J\right)^{-1}\left(J_n-\phi^T\beta^TA\right) \tag{54}$$

$$\sigma'^2_{n,\backslash I} = \sigma_I^2\alpha_I^T\alpha_I-\sigma_I^4\alpha_I^T\phi\left(\sigma_I^2\phi^T\phi+\sigma_J^2\mathbf{I}_J\right)^{-1}\phi^T\alpha_I \tag{55}$$

Fig. 1 shows the shifted dependency structure caused by the marginalization of I.

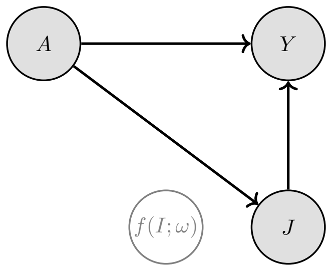

Figure 1. Directed acyclic graph expressing the conditional dependence structure for samples with a missing image I. A are the tabular characteristics, $f(I;\omega)$ are automatically extracted image features, J are manual image measurements, and Y is the outcome. Arrows indicate a dependency, e.g., J only depends on A. By marginalizing over $f(I;\omega)$, a causal link between J and Y is formed.

Missing Data in A *and Missing Image* I

Finally, the case when both the image I and some variables in A are missing. Again we provide the log-likelihood.

$$\begin{aligned}\ln p(A_n^{(\backslash k)}, J_n, Y_n) &= \ln \int\int p(A_n^{(\backslash k)}, A^{(k)}, J_n, f(I_n;\omega), Y_n) dA^{(k)} dI \\ &= \ln p(A_n^{(\backslash k)}) + \ln \mu_{n,\backslash A^{(k)},\backslash I}^{Y_n} (1-\mu_{n,\backslash A^{(k)},\backslash I})^{1-Y_n} + \ln \mathcal{N}(J_n; \phi^T \beta^T \mu_{A'}, \phi^T \left(\beta^T \Sigma_{A'} \beta + \sigma_I^2 \mathbf{I}_I\right) \phi + \sigma_J^2 \mathbf{I}_J)\end{aligned} \tag{56}$$

$$\mu_{n,\backslash A^{(k)},\backslash I} = \sigma\left(\frac{\mu'_{n,\backslash A^{(k)},\backslash I}}{\sqrt{1+\frac{\pi}{8}\sigma'^2_{n,\backslash A^{(k)},\backslash I}}}\right) \tag{57}$$

$$\mu'_{n,\backslash A^{(k)},\backslash I} = \alpha_A^T \mu_{A'} + b_Y + \left(\alpha_A^T \Sigma_{A'} \beta + \sigma_I^2 \alpha_I^T\right) \phi (\phi^T (\beta^T \Sigma_{A'} \beta + \sigma_I^2 \mathbf{I}_I) \phi + \sigma_J^2 \mathbf{I}_J)^{-1} (J_n - \phi^T \beta^T \mu_{A'}) \tag{58}$$

$$\begin{aligned}\sigma'^2_{n,\backslash A^{(k)},\backslash I} &= \alpha_A^T \Sigma_{A'} \alpha_A + \sigma_I^2 \alpha_I^T \alpha_I \\ &\quad - (\alpha_A^T \Sigma_{A'} \beta + \sigma_I^2 \alpha_I^T) \phi (\phi^T (\beta^T \Sigma_{A'} \beta + \sigma_I^2 \mathbf{I}_I) \phi + \sigma_J^2 \mathbf{I}_J)^{-1} \phi^T (\beta^T \Sigma_{A'} \alpha_A + \sigma_I^2 \alpha_I)\end{aligned} \tag{59}$$

References

1. Bishop, C. M. *Pattern Recognition and Machine Learning* (Springer-Verlag, Berlin, Heidelberg, 2006).
